# Response to the letter to the editor regarding “Prediction accuracy of cochlear implant electrode insertion using the OTOPLAN software”

**DOI:** 10.1007/s00405-025-09658-5

**Published:** 2025-08-31

**Authors:** Caterina Vazzana, Marten Geisen, Uwe Baumann, Dennis Sakmen, Esther Knörle, Timo Stöver, Silke Helbig

**Affiliations:** 1 Department of Otorhinolaryngology, Head and Neck Surgery, Goethe University Frankfurt, University Hospital, Frankfurt, Germany; 2 Department of Audiology & Audiological Acoustic, Goethe University Frankfurt, University Hospital, Frankfurt, Germany

Dear Dr. Shafi Ullah,

We would like to thank you for the thoughtful and detailed comments on our study. We appreciate the opportunity to respond and are pleased that our work has generated further discussion on the clinical utility of the OTOPLAN software. Below, we address each of the points raised:

1. Inter-rater reliability

Our study did not include an inter-rater reliability analysis. However, as cited in our manuscript, prior investigations [1, 2] have already demonstrated high inter- and intra-rater agreement for OTOPLAN-based cochlear measurements. Given this well-documented reproducibility and to ensure internal consistency, all measurements in our study were performed by a single experienced CI surgeon. This approach is common in validation studies and does not, in our view, compromise the robustness of our findings.

2. Functional outcomes and predictive use

While we fully agree that auditory outcomes are an important endpoint in cochlear implantation, our study was designed to address a distinct question—namely, the predictive accuracy of OTOPLAN in estimating electrode insertion angle before surgery. Assessing functional outcomes such as speech perception or hearing preservation was outside the scope of this analysis.

The study by O’Connell et al. [3], referenced in the letter, was retrospective and used postoperative imaging to assess angular insertion depth, correlating these values with speech understanding and hearing preservation. Crucially, their study did not involve preoperative prediction or planning based on cochlear measurements. Therefore, it is methodologically unrelated to our study, which evaluates the accuracy of software-based predictions available preoperatively.

Importantly, our own group has previously published data confirming the correlation between deeper insertion angles and improved outcomes in patients with complete deafness [4]. These findings have already begun to inform clinical decisions regarding electrode selection in patients with and without residual hearing and allow for electric versus electric-acoustic stimulation.

In our cohort, OTOPLAN predictions were available preoperatively and actively influenced surgical decision-making. In patients with profound deafness, deeper electrode insertions were often deliberately chosen to achieve full cochlear coverage based on the predicted cochlear dimensions. While this highlights the practical utility of OTOPLAN, it also introduces a potential incorporation bias, as the predicted values directly guided the treatment approach. Other studies that do not consider preoperative cochlear size measurements or OTOPLAN predictions may demonstrate greater variability in speech understanding within their cohorts. This preoperative customization likely reduces variability in outcomes, as electrode selection is tailored to individual anatomy and treatment plans, potentially leading to smaller differences in speech perception results. It would not have been ethically appropriate to ignore known anatomical information for the sake of methodological neutrality. Once available, such data must be used to optimize patient outcomes. The full potential of predictive software such as OTOPLAN is realized especially in patients with residual hearing, where careful planning allows for atraumatic insertions that preserve delicate cochlear structures.

3. Pediatric and anatomically variant cohorts

We excluded pediatric patients for ethical and methodological reasons. Reliable postoperative CBCT imaging in very young children would have required general anesthesia solely for study purposes, which was not justifiable. Furthermore, the cochlea completes its growth and reaches its definitive adult size before birth, so including children would not have introduced significant anatomical variability relevant to the core question of our study.

Cochlear malformations were also excluded, as they represent a separate clinical and anatomical category. These cases are highly heterogeneous and often require individualized surgical approaches. OTOPLAN’s applicability in these settings is of great interest and should be investigated in dedicated studies designed specifically for such non-standard anatomies.

4.  Single-center, retrospective design

We acknowledge that the single-center, retrospective nature of our study limits generalizability. Nonetheless, with 139 cochlear implants analyzed, we believe our data offer meaningful insight into the performance of predictive imaging software in clinical routine. It is important to note that by measuring preoperative data, one could essentially achieve the same situation as in a prospective study, where predictions are made beforehand and later compared with outcomes. We support prospective, multicenter studies as the next step toward broader validation.

5.  Imaging parameters and scanner consistency

All imaging in our study was performed using the same CBCT scanner, with standardized protocols and thin-slice reconstruction. This ensured a high level of internal consistency and eliminated variability related to scanner model or imaging settings. While we agree that differences in scanner technology and reconstruction algorithms could influence cochlear metrics, such a comparison would require a multicenter design and was beyond the scope of the present investigation.

We thank the authors once again for their constructive feedback. While many of the points raised were addressed in our original manuscript, we agree that they highlight important directions for future research. We hope our findings contribute to the continued development and validation of individualized, imaging-based planning tools for cochlear implantation.

Sincerely,

Silke Helbig and Caterina Vazzana

On behalf of all authors
